# Biological Significance of Tumor Heterogeneity in Esophageal Squamous Cell Carcinoma

**DOI:** 10.3390/cancers11081156

**Published:** 2019-08-12

**Authors:** Lehang Lin, De-Chen Lin

**Affiliations:** Guangdong Provincial Key Laboratory of Malignant Tumor Epigenetics and Gene Regulation, Medical Research Center, Sun Yat-Sen University, Guangzhou 510120, China

**Keywords:** esophageal squamous cell carcinoma, tumor heterogeneity, tumor evolution, precision medicine

## Abstract

Esophageal squamous cell carcinoma (ESCC) is a common and aggressive malignancy, with hitherto dismal clinical outcome. Genomic analyses of patient samples reveal a complex heterogeneous landscape for ESCC, which presents in both intertumor and intratumor forms, manifests at both genomic and epigenomic levels, and contributes significantly to tumor evolution, drug resistance, and metastasis. Here, we review the important molecular characteristics underlying ESCC heterogeneity, with an emphasis on genomic aberrations and their functional contribution to cancer evolutionary trajectories. We further discuss how novel experimental tools, including single-cell sequencing and three-dimensional organoids, may advance our understanding of tumor heterogeneity. Lastly, we suggest that deciphering the mechanisms governing tumor heterogeneity holds the potential to developing precision therapeutics for ESCC patients.

## 1. Introduction

Esophageal carcinoma is the sixth most lethal cancer type worldwide, responsible for over 400,000 deaths annually [1,2]. Esophageal squamous cell carcinoma (ESCC) is the predominant histological subtype, accounting for 90% of cases [3,4]. Despite noteworthy advances in both cancer diagnosis and therapy, the clinical outlook for ESCC patients remains dismal, with a five-year survival rate below 30% [5,6]. A number of lines of evidence have demonstrated that this poor clinical outcome is at least partially attributed to the substantial intertumor and intratumor heterogeneity in ESCC [7,8].

The concept of tumor heterogeneity contains both intertumor and intratumor forms. Intertumor heterogeneity concerns the phenotypic and molecular differences among tumors from different patients, while intratumor heterogeneity refers to biological variations within the same tumor [9,10,11,12,13]. Heterogeneity is an important attribute of cancer and a major contributor to tumor progression. It manifests at two major levels: genomic (somatic mutations, copy number alterations, chromosomal rearrangements, etc.) and non-genomic (epigenomic changes, microenvironmental variabilities, etc.) [14,15]. The degree and complexity of tumor heterogeneity influence the strategy of tumor biopsy, cancer diagnosis, and treatment planning [7,9,14,16,17,18]. Increasingly, advances in sequencing technology and analysis algorithms have substantially promoted the understanding of both intertumor and intratumor heterogeneity in many cancer types, including ESCC [7,19,20]. However, translation of the accumulated knowledge on ESCC heterogeneity into clinical practice is still challenging. A systematic understanding of ESCC heterogeneity with respect to its composition, function, and implication is therefore urgently needed.

In this review, we summarized the evidence for both genomic and non-genomic sources of ESCC heterogeneity and discussed their biological and clinical significance in the context of tumor evolution. We also described new technologies and methodologies that might further our understanding and management of ESCC heterogeneity and shared our perspectives on the future of this field.

## 2. Intertumor Heterogeneity

Taxonomy of cancer subtypes by specific molecular characteristics significantly improves the conventional histopathological classification and guides subtype-specific precision medicine. As exemplified in breast cancer (e.g., luminal, basal-like, Her2+), lung cancer (e.g., EGFR+, ALK fusion+), and gastric cancer (e.g., Epstein–Barr virus+, microsatellite unstable), intertumor heterogeneity has been widely studied and successfully translated into clinical knowledge in various cancer types [21,22,23]. However, the stratification of ESCC patients based on intertumoral molecular heterogeneity remains comparatively understudied.

In 2017, through an integrative multi-omics analysis, The Cancer Genome Atlas (TCGA) consortium classified 90 ESCC specimens into three subtypes, designated as ESCC1–3 [4]. ESCC1, mostly Asian samples, was enriched in genomic alterations in the *NRF2* pathway (*NFE2L2, KEAP1, CUL3,* and *ATG7*) and amplifications of *SOX2* and/or *TP63*; ESCC2, mainly Eastern European and South American samples, was characterized by higher rates of *NOTCH1* and *ZNF750* mutations, *CDK6* amplification, and inactivation of *KDM6A*, *KDM2D*, *PTEN*, and *PIK3R1*; only four cases were classified into ESCC3, which were all from North America and featured in *SMARCA4* mutation. Although these subtypes showed notable geographical trends, their associations with particular biological and/or clinical features were not extensively elucidated. In addition, because of the relatively small number of samples, these classifications need further validation in larger cohorts.

In addition to the effort from TCGA consortium, several individual laboratories have attempted to subgroup ESCC based on transcriptomic data. Upon analyzing tumor samples from African patients, Liu et al. [24] reported three ESCC subtypes based on their distinct expression patterns of cell cycle and neural transcripts. In another study, ESCC specimens from 360 East Asian individuals were divided into four molecular subtypes associated with distinct clinical metrics [25]. Most recently, a new research work has categorized Asian ESCCs into two subtypes, with subtype I overexpressing genes in immune response process and subtype II linked to ectoderm development, cell proliferation, and glycolysis process [26]. Additionally, Tanaka et al. [27] reported the presence of an immune-reactive subtype of ESCC patients with cytotoxic T-lymphocyte signatures activated by chemoradiotherapy.

Despite the fact that no consensus molecular subtypes of ESCC have been established, the above subtyping results are sufficient to confirm the existence of extensive intertumor heterogeneity among ESCC individuals, and further demonstrate heterogeneity amongst different ESCC ethnic groups. This is in line with the well-established dramatic geographic and demographic features of ESCC [28,29]. It should also be noted that the molecular factors and causes underlying intertumor heterogeneity are likely similar with those involved in intratumor diversity. In order to fully capture the tumor spectrum, and to further improve ESCC subclassification and treatment stratification, the molecular features of ESCC intratumor heterogeneity need to be comprehensively integrated.

## 3. Intratumor Heterogeneity

**Figure 1:**
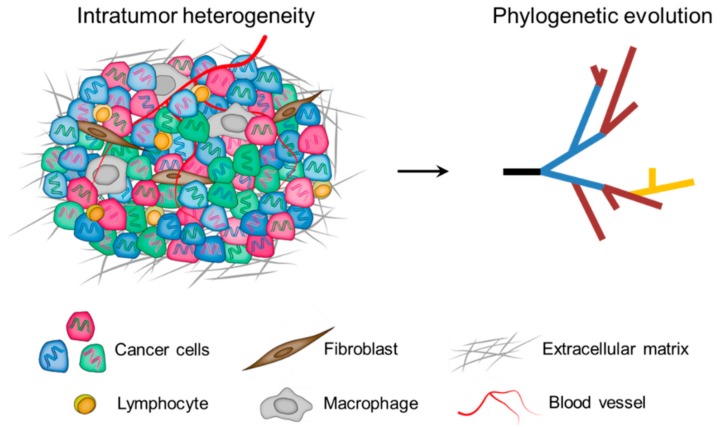
Multiple layers of intratumor heterogeneity. **Left**: Phenotypically distinct cancer cells with both genomic (DNA color) and epigenomic (cell color) heterogeneity are admixed with diversified microenvironmental components. **Right**: A phylogenetic framework helps to understand the nature and biological significance of tumor spatiotemporal heterogeneity.

In the milestone paper published in 1976, Peter C. Nowell [30] proposed a model for cancer development: the Darwinian clonal evolution and selection of tumor cells. Since then, this model has been widely accepted and the phenomenon of intratumor heterogeneity has been highlighted as a cancer hallmark to reflect the non-uniformity and intricacy within tumor ecosystems [31,32,33]. To date, it is well established that intratumor heterogeneity is represented by the presence of distinct cell populations, which can occupy specific microenvironmental niches, behave as communities, and extensively interact with each other as well as with components of the tumor microenvironment [12]. Therefore, intratumor heterogeneity arises not only from genomic and epigenomic disorders of tumor cells themselves, but also from the influence of the tumor microenvironment [9]. Importantly, intratumor heterogeneity exists among different geographical regions of the same tumor (spatial heterogeneity), as well as between the primary tumor and subsequent local or distant recurrence in the same patient (temporal heterogeneity). As a cumulative result, tumor cells display remarkable variability in numerous phenotypic traits, including clinically important phenotypes such as the ability to seed metastases and to survive therapy (Figure 1) [34].

### 3.1. Clonal Evolution of Tumors

According to the clonal evolution hypothesis, cancer arises from a single founder cell, and tumor progression is accompanied by the resultant succession of clonal expansions that follow the Darwinian logic [30]. This evolutionary perspective underlines genomic alterations as an essential substrate for fueling tumor transformation and evolution.

During each cell cycle, regardless of normal or cancer cells, DNA mutations may be acquired. Thus, the acquisition of mutations is a stochastic and random process. Consequently, innumerable rounds of cell divisions required for the formation of macroscopic tumors offer plenty of opportunities for Darwinian selection and emergence of clonal diversity in tumor cell populations. During clonal evolution, only a few “jackpot” mutations that activate oncogenic pathways and/or inactivate tumor suppressors are selectively advantageous, allowing the mutant clones to achieve selective sweeps. These functionally significant mutations are termed “drivers”. In contrast, the vast majority of mutations are functionally neutral since they do not confer competitive fitness advantage. These mutations are so-called “passengers” and are mainly responsible for intratumor heterogeneity [35]. Importantly, clonal evolution often proceeds in a branching rather than in a linear manner, further contributing to variegated tumor subclones and the complexity of tumor evolution [14]. In fact, many neutral or mildly deleterious mutations during clonal expansion can be retained in the population, or even undergo expansions due to the genetic drift [32,35]. Moreover, given the fact that the Darwinian selection is context-specific, and the evolutionary dynamics of tumor microenvironment and epigenomic events could translate into heterogeneous selective pressures experienced by tumor cells, the selective effect of given mutations (either driver or passenger) can change substantially at different stages of tumor progression [14].

### 3.2. Spatial Intratumor Heterogeneity

Spatial intratumor heterogeneity has been elucidated at high resolution in many cancer types [15,36,37,38,39,40]. Recently, several groups have performed multi-regional deep-sequencing, and have presented a comprehensive heterogeneous landscape of ESCC [41,42,43,44]. Through analyzing 51 sub-tumor regions from 13 ESCC patients, Hao et al. [42] proposed that approximately 40% of driver mutations were spatially heterogeneous, including oncogenes such as *KIT*, and members of the *PI3K*/*MTOR* (*PIK3CA* and *MTOR*) and *NFE2L2* pathways (*NFE2L2* and *KEAP1*). In addition, significant spatial heterogeneity was observed in copy number alterations, including *EGFR* amplification and *CDKN2A/B* deletions [42]. Furthermore, taking into consideration the multi-step progression of ESCC, Zeng’s team [43] sequenced different segments of ESCC tumors and their matched dysplasia samples in a cohort of 20 patients. Their analyses showed that esophagus dysplasia also carried high mutation load and, remarkably, more heterogeneous mutations were seen in dysplasia than in tumor samples from each patient. Moreover, through sequencing 682 micro-scale esophageal samples, Yokoyama et al. [45] reported very recently that pervasive expansions of multiple independent clones were more commonly present within physiologically normal esophagus in comparison to ESCCs. These seemingly surprising data indicate that diversified mutational backgrounds were already established in the precursor lesion or even normal esophageal epithelia, conferring on the esophageal cells the ability to evade selection pressure during ESCC development. Moreover, the degree and complexity of spatial heterogeneity was found to be highly correlated with ESCC aggressiveness [44]. Specifically, clinical stage of ESCC was negatively correlated with the proportion of ubiquitous mutations, and significantly more heterogeneous mutations were observed in ESCC patients with local metastasis, compared to those without.

Regionally segregated somatic mutations and copy number alterations have important clinical implications in ESCC. Firstly, they complicate pathological evaluation of tumor samples. Owing to potential sampling bias caused by spatial heterogeneity, the representability of tumor regions subject to pathological assessment is increasingly considered as a key factor. It is possible that diagnostic and therapeutic targets located in uninspected regions are missed by chance, and the heterogeneous spectrum of the tumor is inevitably underestimated. Additionally, spatial genomic heterogeneity is an important determinant for therapeutic responses. Although most cancers initially respond to treatment, they almost always relapse with the outgrowth of cancer cells that are no longer sensitive to the therapy. Many cases have demonstrated that resistance to targeted drugs may result from the preexisting heterogeneous cells. Examples include the impaired efficiency of EGFR inhibitor for lung cancer patients with heterogeneous driver status [46,47]. Lung cancers initially containing rare mutations of *EGFR*, e.g., T790M, or low frequency of *MET* amplification, are capable of rendering resistance to targeted therapy [18,31,48,49]. Another well-understood case is chronic myeloid leukemia, in which mutant forms of the BCR-ABL fusion protein have been implicated in the relapse of disease under imatinib treatment [50,51,52]. In ESCC, heterogeneous amplifications of *EGFR*, *FGFR1*, and *PD-L1* have been reported [42,43,44], accounting partially for the unsatisfactory efficacy of targeting such genomic lesions [53,54,55]. Spatial genomic heterogeneity, therefore, greatly challenges both accurate diagnosis and efficient cancer treatment.

In addition to genomic alterations, epigenomic dysregulation also contributes to spatial diversity within a tumor. Mechanistically, epigenomic heterogeneity may arise from changes in chromatin status (e.g., DNA methylation, histone modification), deregulation of microRNAs, and transcription regulators, etc. These alterations potentially provide fitness benefit, leading to intratumor heterogeneity either independently or in conjunction with genomic alterations [56,57,58,59,60,61,62,63,64]. For example, DNA methylation status within promoters of transcription factors SIM2 and SIX1 was strongly correlated with their heterogeneous expression pattern, which was further associated with ESCC differentiation, progression, and prognosis [65,66,67]. Dynamic changes of mutational status and promoter DNA methylation were also observed in the SWI/SNF chromatin remodeling complex and were shown to involve in ESCC carcinogenesis [68]. Moreover, epigenomic and genomic heterogeneity have been integratively analyzed in three ESCC patients [42]. Noticeably, the spatial heterogeneous pattern of DNA methylation closely recapitulated that of somatic mutations, indicating functional interplay between genomic and epigenomic alterations in ESCC.

The tumor microenvironment, consisting of fibroblasts, extracellular matrix, immune cells (e.g., macrophages, infiltrating lymphocytes), etc., imposes yet another layer of heterogeneity [44,69,70,71,72,73,74,75]. Tumor microenvironment can shape tumor cell phenotypes by augmenting both the intrinsic variability of cancer cells (e.g., by inducing stress responses and genomic instability) and the extrinsic diversity of microenvironmental contexts (e.g., different densities of blood and lymphatic vasculature, different numbers and types of infiltrating cells) [7]. In ESCC, the tumor microenvironment itself is indeed highly heterogeneous, as evidenced by recent reports of intratumor heterogeneity of tumor infiltrating T and B cells [44,70,75]. Additionally, Yan et al. [44] observed a tight association between genomic heterogeneity and variation of T cell repertoire in ESCC primary tumors. These results demonstrate that the intratumor genomic heterogeneity may have clinical relevance in ESCC through affecting tumor microenvironment. Meanwhile, ESCC cells could also benefit from the microenvironmental heterogeneity, which supports cellular diversity and influences evolutionary trajectories [14,62,76,77].

### 3.3. Temporal Intratumor Heterogeneity

Accumulating evidence suggests that intratumor heterogeneity contributes to tumor growth through a process called branched evolution. This model suggests that tumorigenesis is analogous to a growing tree, whose trunk gives rise to numerous branches [9,14,78]. Phylogenetic analysis is a useful approach to delineate such tree structure of cancer evolution [19,38,79,80,81]. Accordingly, in the phylogenetic tree, truncal (ubiquitous) events shared by the entire tumor population likely reflect processes involved before and during tumor initiation and early development, whereas branched (heterogeneous) events present in only some regions of the tumor reveal factors shaping the genome during tumor maintenance and progression. Characterization of the relative timing of key somatic events with possible biological relevance is therefore essential for deciphering the evolutionary processes of tumors, as well as further improving precision medicine strategies.

In ESCC, driver mutations were significantly more truncal/clonal than passenger mutations, in accordance with findings in other tumor types. Importantly, the majority of driver mutations in tumor suppressors (including *TP53*, *KMT2D*, *ZNF750*, etc.) had a tendency to locate in the trunks of phylogenetic trees, indicating that tumor suppressors are lost as relatively early events during ESCC development. In contrast, half of the driver mutations in the branches were in oncogenes, including potential actionable targets, *PIK3CA* and *MTOR*, suggesting that they are late events in ESCC [42]. This observation highlights the extra caution needed when considering inhibiting such oncogenic mutants in ESCC, given previous studies showing that suppressing subclonal drivers could otherwise lead to outgrowth of non-mutated subpopulations [82].

Esophageal squamous cell carcinoma evolution is a multi-step process that begins from low-grade dysplasia, high-grade dysplasia, carcinoma in situ to invasive tumor and metastasis [44]. To further explore the genomic dynamics during this process, recent studies applied multi-region sequencing on samples covering different stages of ESCC from the same patients and constructed phylogenetic trees that mapped mutations and copy number alterations chronologically [43,44,83]. Notably, only a small fraction of total genomic alterations was conserved from squamous dysplasia to ESCC tumors, implying the distinct evolutionary trajectories taken by precursor and neoplastic cells [43,83]. Phylogenetic analysis confirmed truncal mutations of *TP53* and *CDKN2A* and truncal copy number alterations of 11q13 (*CCND1*), 3q27 (*SOX2*), 2q31 (*NFE2L2*), and 9p21 (C*DKN2A*), validating that they are early changes during esophagus neoplastic transformation [83]. Independently, Chen et al. [43] also reported early emergence of copy number alterations in precursor lesions of ESCC and highlighted this phenomenon as a prominent genomic feature distinct from the development of esophageal adenocarcinoma, another pathological subtype of esophageal cancer. When considering alterations at pathway level, genes involved in cell cycle regulation (such as *TP53*, *CCND1*, *CDK6*, *RB1*, and *CDKN2A*) were frequently altered in the early stage of ESCC, whereas genes in *RTK*/*RAS*/*PI3K* tended to undergo alterations throughout the process of ESCC evolution [44].

**Figure 2:**
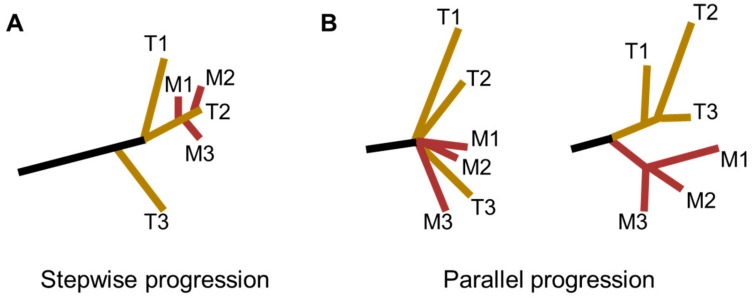
Phylogenic models for ESCC metastasis. (**A**) The stepwise progression model, in which metastases are seeded at the late stages of ESCC progression. This model was also described as the linear spread pattern by Yan et al. [44]. (**B**) The parallel progression model, in which tumor dissemination occurs at the early stages of ESCC progression. In this situation, dissemination of metastases could happen either (**Left**) explosively (explosive spread pattern) or via (**Right**) metastasis-to-metastasis pattern (T, Tumor; M, Metastasis).

Taking into consideration of the timing of metastatic outgrowth and the role of the intratumor heterogeneity, two distinct models for the derivation of ESCC metastasis have been proposed: the stepwise progression model and the parallel progression model (Figure 2) [43,44]. The stepwise progression model was characterized by tumor cells disseminated at the late stages of ESCC. Accordingly, metastases could be considered as direct descendants of the most malignant and aggressive clones that dominated primary tumors. This model was also described as the linear spread pattern by Yan et al. [44]. By comparison, in the parallel progression model, early spread of metastases during ESCC tumor progression was highlighted. Specifically, divergent evolutionary trajectories were found between primary tumors and metastatic lesions, as well as among metastatic lesions. This model was represented as both explosive spread and metastasis-to-metastasis patterns by Yan and colleagues [44].

More studies are required to elucidate the clonal relationship between ESCC primary and metastatic tumor cell populations, which will not only illuminate the evolutionary history of ESCC, but also create a more solid ground for therapeutic decision making. Ultimately, decoding the extent of differences between ESCC primary and metastases is crucial for the improved management of metastatic ESCC patients.

## 4. New Technologies for the Investigation of Intratumor Heterogeneity

It is with great excitement that we are witnessing novel technologies and methodologies being developed at a fast pace for the investigation of intratumor heterogeneity. These advances will uncover the molecular and biological features of intratumor heterogeneity with unprecedented resolution and they hold the potential to revolutionize our understanding of the complex intrinsic and extrinsic heterogeneity of ESCC.

### 4.1. Heterogeneity Studies at the Single-Cell Level

Technical advances in single-cell sequencing have been heralding a new era in resolving tumor heterogeneity and understanding the dynamics of subclonal architecture during tumor progression [31,36,84,85,86,87,88]. For instance, single-cell RNA sequencing of glioblastomas revealed that cell-to-cell inherent variability was evident in regulatory axes central to glioblastoma biology, prognosis, and therapy [88]. Single-cell DNA sequencing of a large number of breast cancer cells unraveled the punctuated evolution pattern of copy number alterations during tumor development [36,86,87]. In ESCC, single-cell RNA sequencing has been recently performed on three specimens, and substantial intratumor heterogeneity contributed by both tumor-intrinsic and microenvironmental alterations has been observed [89]. Nevertheless, more extensive single cell-based studies of ESCC are still lacking.

Compared to conventional bulk-tissue analysis, single-cell profiling is superior in its robust sensitivity in detecting subclonal/private genomic alterations at true single-cell resolution. Thus, single-cell sequencing is of pivotal importance in discovering subtle diversification and rare tumor clones. Looking to the future, we anticipate that the application of single-cell multi-omics technologies (e.g., transcriptome, methylome [90,91], and chromatin [92,93] assays) to both cancerous cells and infiltrating stromal cells will finally provide us with a panoramic view of ESCC heterogeneity.

### 4.2. Three-Dimensional Organoid Culture

Organoid cultures are three-dimensional multicellular constructs wherein cells can self-assemble to faithfully represent the physiological states of parent tissues or organs. Derived from patients, 3D organoids are crucial tools for disease modeling, especially cancers. Briefly, the generation of patient-derived organoids involves disintegration or digestion of the tumor tissue into single-cell suspensions or cell aggregates, followed by implantation of the cells in growth factor-optimized media and 3D basement membrane matrix (Matrigel) [94]. Currently, 3D organoids have been developed for several cancer types and been proven to successfully recapitulate tumor spatiotemporal heterogeneity [95,96,97,98,99,100]. Particularly, the generation of clonal organoids from different locations from the same tumors allows the study of tumor heterogeneity and evolution [100,101,102,103]. Specific driver and passenger mutations could be introduced by the CRISPR-Cas9 genome-editing system with the resultant organoids valuable for dissecting tumor progression mechanisms [104,105,106,107]. Importantly, organoid technology has also been leveraged to facilitate the development of personalized medicine, including drug screening and therapy response evaluation, which is in urgent need to overcome tumor heterogeneity [98,101,108,109,110,111,112].

In ESCC, Kijima and colleagues [113] have successfully generated and characterized 3D organoids from ESCC patients and provided proof-of-principle regarding their utility as a robust platform for analyzing cancer cell heterogeneity, evaluating drug sensitivity, and exploring mechanisms of drug resistance. However, given the importance of the tumor microenvironment, future work will require the investigation of ESCC-relevant niche factors and optimization of conditions for co-culturing with stromal and immune cells in the 3D organoids [114]. It also needs to be determined whether the 3D organoids could faithfully recapitulate the entire heterogeneity of ESCC, and to what extent could the heterogeneity be maintained after extended ex vivo culture. Additionally, future applications of 3D organoids in the clinical setting should include analysis of both ESCC precancerous and metastatic lesions. With such improvements in the organoid system, we believe that 3D organoid culture will become a powerful tool to model the biological function of intratumor heterogeneity. Through this tool, we can gain more insights into ESCC evolution in time and space and develop more effective precision medicine against this deadly disease.

## 5. Conclusions and Future Directions

It is now clear that heterogeneity is a hallmark of ESCC. However, a significant lag still exists between the knowledge advance of tumor heterogeneity and its clinical translation regarding to diagnostic, prognostic, and therapy. Firstly, it is worth investigating whether the heterogeneity degree by itself could serve as an important biomarker in predicting ESCC progression and guiding treatment decisions. Notably, the extent of clonal diversity predicts the probability of malignant progression in Barrett’s esophagus [115]. The quantitative evaluation of cellular diversity has also been used as a biomarker in breast cancer [116]. These examples suggest that diagnostic value of heterogeneity may have universal applicability in cancers irrespective of tumor anatomy. Secondly, given that a heterogeneous tumor is the resulting accumulation of both genomic and non-genomic diversities, the heterogeneity of both cellular and non-cellular components (e.g., epigenome, tumor microenvironment) should be more extensively explored, together with their associations with ESCC biology and clinical outcomes. This entails chronological studies assessing heterogeneity along disease progression of the same patient, from squamous dysplasia to cancer metastases. Moreover, longitudinal studies evaluating intratumor heterogeneity changes during and following therapeutic interventions are required to support identifying early resistant disease and limiting iatrogenic impacts of therapy on ESCC evolution. Thirdly, although heterogeneity undoubtedly poses a formidable obstruction to therapeutic success, there exists a potential to reach drug-sensitive states by strategies of reducing or even exploiting tumor heterogeneity. Current promising therapeutics include targeting epigenetic modifications of cancer cells (e.g., histone deacetylase inhibitors), modulating the tumor microenvironment (e.g., anti-angiogenic therapy, immune therapy), as well as multiplex-targeted therapy and adaptive therapy. Importantly, there is also a significant progress in the development of anti-cancer stem cell (CSC) treatments, since CSCs constitute a new research focus in tumor heterogeneity [117,118,119]. Particularly, CSCs not only contribute to the phenotypic heterogeneity of primary tumor but can persist under treatment and provoke drug-resistant recurrence and distant metastasis. In conclusion, advanced theoretical understanding of the tumor heterogeneity along with the rapid development of associated technologies will help develop more innovative and effective precision medicine against ESCC.
